# Modulation of Fecal Metabolites by Heat Stress and Diet, and Their Association with Inflammation and Leaky Gut Markers in Dairy Cows

**DOI:** 10.3390/metabo12020142

**Published:** 2022-02-03

**Authors:** Alexis Ruiz-González, Daniel Enrique Rico, Jorge Eduardo Rico

**Affiliations:** 1 Department of Animal Science, Université Laval, Quebec City, QC G1V 2L2, Canada; alexis.ruiz-gonzalez.1@ulaval.ca; 2 Centre de Recherche en Sciences Animales de Deschambault (CRSAD), Deschambault, QC G0A 1S0, Canada; 3 Department of Animal and Avian Sciences, University of Maryland, College Park, MD 20742, USA

**Keywords:** heat stress, fecal metabolites, vitamin D_3_, vitamin E

## Abstract

The analysis of fecal metabolite profiles could provide novel insights into the mechanisms underlying animal responses to environmental stressors and diet. We aimed to evaluate the effects of a 14-day heat stress period and of dietary mineral and vitamin supplementation under heat stress on fecal metabolite profiles and to investigate their associations with physiological markers of heat stress, leaky gut, and inflammation in lactating dairy cows. Twelve multiparous Holstein cows (42.2 ± 5.6 kg milk/d; 83.4 ± 27.1 DIM) were enrolled in an experiment in a split-plot design. The main plot was the level of dietary vitamin E and Se, as follows: (1) low (L-ESe; 20 IU/kg vitamin E, 0.3 ppm Se) or (2) high (H-ESe 200 IU/kg vitamin E, 1.2 ppm Se). Within each plot, six cows were randomly assigned to either (1) heat stress (HS; Total Humidity Index (THI): 82), (2) pair-feeding in thermoneutrality (TNPF; THI = 64), or (3) HS with vitamin D_3_ and Ca supplementation (HS+DCa; 1820 IU/kg and 1.5% Ca; THI: 82) in a replicated 3 × 3 Latin square design with 14-day periods and 7-day washouts. The concentrations of 94 metabolites were determined in fecal samples, including amino acids, fatty acids, biogenic amines, and vitamins. Relative to the L-ESe group, the H-ESe group increased α-tocopherol by threefold, whereas δ-tocopherol was decreased by 78% (*P*_FDR_ < 0.01). Nevertheless, correlation analysis between α-tocopherol and all the others fecal metabolites or physiological heat stress measures did not show significant associations. No interactions between main plot and treatments were observed. Relative to TNPF, HS increased plasma tumor necrosis factor-alpha (TNF-α), plasma lipopolysaccharide-binding protein (LBP), milk somatic cell counts (SCC), respiratory rates, rectal temperatures, fecal tridecylic and myristic acids, vitamin B_7_, and retinol, whereas it decreased fecal amino acids such as histidine, methyl histidine, acetyl ornithine, and arginine (*P*_FDR_ < 0.05). In contrast, HS+DCa increased fecal methyl histidine concentrations and reduced milk SCC, plasma TNF-α, and LBP, as well as rectal temperatures. Discriminant analysis revealed fecal histidine, taurine, acetyl ornithine, arginine, β-alanine, ornithine, butyric + iso-butyric acid, plasma non-esterified fatty acids, TNF-α, LBP, C-reactive protein, and milk SCC were predictive of HS. Several metabolites were predictive of HS+DCa, although only tryptophan was discriminant relative to HS. In conclusion, both heat stress and the supplementation of vitamin D_3_ and Ca can influence the fecal metabolome of dairy cows experiencing heat stress, independently of dietary levels of vitamin E and Se. Our results suggest that some fecal metabolites are well associated with physiological measures of heat stress and may thus provide insights into the gut-level changes taking place under heat stress in dairy cows.

## 1. Introduction

Global warming is a major concern for the sustainability of agricultural production as it negatively impacts animal comfort, health, and performance [1,2,3]. Cows under heat stress experience major reductions in appetite, which partly explains the reductions in lactation performance [4,5]; however, changes in gut health and the activation of the immune system also contribute to this phenotype [6,7]. Importantly, the simultaneous reduction in intake of key nutrients and increased nutritional needs to sustain the immune response may hinder the animal’s ability to cope with heat stress challenges.

Gastrointestinal microbiota interacts with the host during both stress and homeostatic conditions [8]. Under homeostatic conditions, the gut microbiota regulate the release of cytokines and chemokines from gut mucosal immune cells, which in turn aid the host maintaining stable bacterial communities in the gut [9]. On the other hand, changes in microbiota composition could also result in immune system activation [8] in cases when intestinal barrier integrity is compromised, such as heat stress, and systemic inflammation. High ambient temperatures and humidity result in increased expression of proinflammatory mediators, such as TNF-α [10,11,12]. This is thought to be the result of acute intestinal ischemia, leading to leaky gut, which in turn allows for permeation of bacterial-origin antigenic molecules such as lipopolysaccharide (LPS) into circulation [13]. Importantly, dietary nutrients, such as vitamin D_3_ and Ca can modulate the immune response under similar scenarios of leaky gut induction in rodents [14], while others such as vitamin E and Se were shown to improve the integrity of the gut lining while reduce oxidative stress under heat stress [15]. Strategic supplementation of such vitamins and minerals may thus mitigate the impact of HS in dairy cows; however, these strategies have not been specifically investigated to date.

While environmental parameters such as temperature and humidity can be used to predict heat stress in farms, they are not direct measures of metabolic changes in dairy cows as a response to HS. In addition to environmental measures (e.g., temperature and humidity [1]), physiological parameters, such as body temperature measures (i.e., rectal, vaginal, or skin) and respiratory rates, can also be used to diagnose heat stress. However, these indicators are highly variable, as they could be influenced by physiological stage (e.g., lactating vs. non-lactating cows) and health status (e.g., mastitis and metritis; [16]). In addition to physiological parameters, indirect markers of heat stress may also have diagnostical value, and can simultaneously improve our understanding of the changes taking place, as they could be associated to an altered metabolic and physiological status.

Some studies have reported changes in the blood and milk metabolite profile after periods of heat stress [17,18]. The comprehensive analysis of fecal metabolites can provide direct insight into the interactions between environment challenges, diet, and the gut-microbiome [19]. In agreement with this possibility, others have reported that following an infection, the host can adapt its metabolism to favor the release of nutrients into the intestinal lumen (e.g., taurine), which can then be used by gut microbiota to fight future pathogen infections [20]. Although these types of mechanisms could be at play, they have not been specifically investigated during heat stress in lactating dairy cows. Furthermore, to our knowledge, no studies in dairy cows have specifically investigated the association between physiological and metabolic changes derived from heat stress (e.g., respiratory rates, rectal temperature, inflammation markers) and fecal metabolites. These investigations could provide valuable insight into potential strategies to mitigate the impact of heat stress on cow productivity and health.

Similar to the effects of environment, diet composition could also directly or indirectly alter the fecal metabolite profile. For instance, high doses of dietary vitamin D_3_ can modulate gut microbiota composition [21], potentially altering fecal metabolites as well. In this manner, the fecal metabolome may be the reflection of both endogenous losses [22], as well as of alterations of the gut microbiota composition [23].

We hypothesized that the ensemble of changes caused by heat stress and dietary nutrients will alter the profile of select fecal metabolites such as amino acids, lipids, and vitamins. In addition, we hypothesized that the physiological changes caused by heat stress, such as leaky gut and inflammation, will be associated to changes in the profile of select fecal metabolites.

The objectives of the present study were twofold: (1) to evaluate the effects of dietary supplementation of mineral and vitamin supplementation on select fecal metabolites under heat stress, and (2) to investigate the associations between physiological indicators of heat stress and fecal metabolites in order to evaluate their potential as biomarkers.

## 2. Results

### 2.1. Fecal Metabolites Concentration

**Figure 1:**
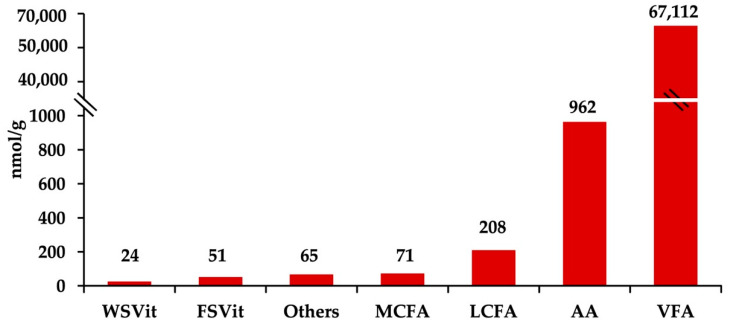
Concentrations of predominant fecal metabolites (nmol/g of fresh feces) in dairy cows across treatments. From right to left: total volatile fatty acids (VFA, <6C), total amino acids (AA), long-chain fatty acids (LCFA, ≥16C), medium-chain fatty acids (MCFA, 6C−15C), other metabolites (choline, asymmetric dimethylarginine, taurine, trimethylamine N-oxide, cystathionine, proline-betaine, caffeine, and trigolline), fat-soluble vitamins (FSVit), and water-soluble vitamins (WSVit). Treatments were: (1) heat stress; (2) heat stress with higher vitamin D_3_ and Ca (1820 IU/kg and 1.5% Ca); (3) thermoneutral, pair-feeding. Treatments were allocated within two plots differing in basal concentrations of vitamin E and Se (high: 200 IU/kg vit. E and 1.2 mg/kg Se; low: 20 IU/kg vit. E and 0.3 mg/kg Se). Fecal metabolite data were obtained using a LC–MS/MS custom assay.

The reverse-phase LC–MS/MS assay identified 94 fecal metabolites including volatile fatty acids, amino acids, fat- and water-soluble vitamins, and medium- and long-chain fatty acids (Supplementary Material Table S1). Figure 1 shows fecal metabolites grouped by affinity. Volatile fatty acids (VFA) exhibited the highest molar proportions of the metabolites detected in fecal samples (67,112 nmol/g; 97.98%), followed by amino acids (AA; 962 nmol/g; 1.40%), long-chain fatty acids (LCFA; 208 nmol/g; 0.30%), medium-chain fatty acids (MCFA; 71 nmol/g; 0.10%), fat-soluble vitamins (FSVit; 51 nmol/g; 0.07%), and water-soluble vitamins (WSVit; 24 nmol/g; 0.04%).

The highest concentrations of essential amino acids (Figure S2a) were observed for lysine (Lys; 32%), valine (Val; 16%), leucine (Leu; 15%), and threonine (Thr; 10%), followed by phenylalanine (Phe; 9%), isoleucine (Ile; 8%), and methionine (Met; 6%). The group of nonessential amino acids (Figure S2b) were represented by alanine (Ala; 30%), glutamate (Glu; 28%), aspartate (Asp; 16%), glycine (Gly; 11%), and serine (Ser; 7%). Fecal short chain fatty acids < 6C (Figure S3a) were represented by acetate (2:0, 88% of total volatile fatty acids; VFA), propionate (3:0, 2%), and butyrate (4:0; 9%) acids. We found that fecal medium-chain fatty acids 6C–15C (Figure S3b) were mainly represented by myristic (14:0) with 54%, followed by lauric (12:0; 18%) and tridecylic acids (13:0; 8%). The highest concentrations of fecal long-chain fatty acids (>16C; Figure S3c) were observed for oleic (18:1, *cis*-9; 47%), palmitic (16:0; 38%), and stearic acids (18:0; 12%). Furthermore, compounds such as p-hydroxyphenylacetic acid (p-HPHA) and benzoic acid were predominant among the remaining metabolites (Figure S4).

### 2.2. Effects of Heat Stress and Vitamin D_3_ and Ca Supplementation during Heat Stress on Fecal Metabolites and Physiological Parameters

**Figure 2:**
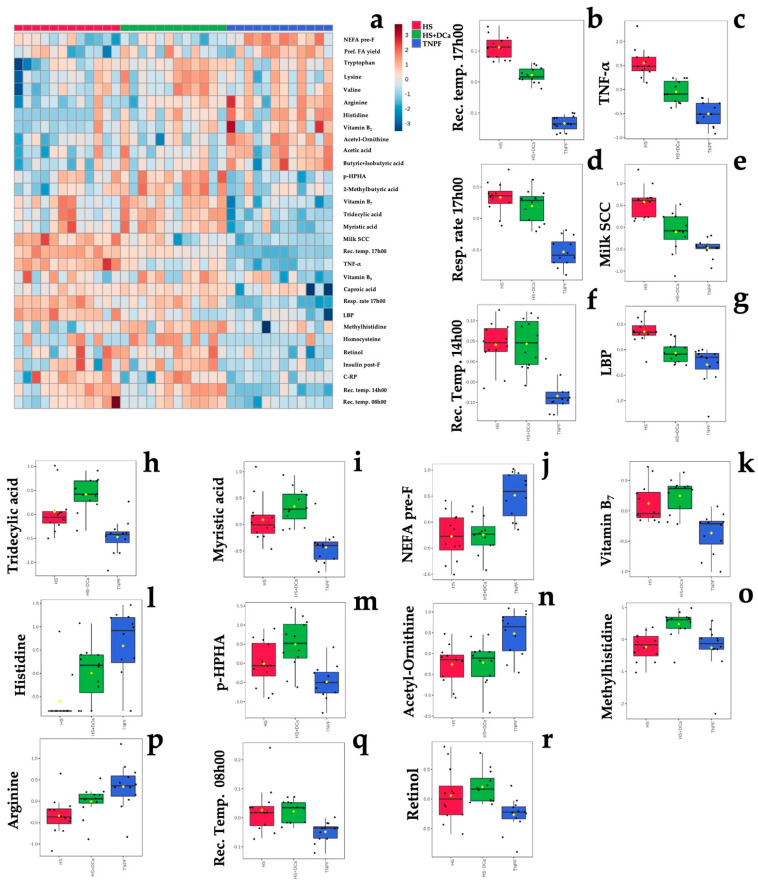
Differences in fecal metabolites of dairy cows following heat stress, heat stress with vitamin D_3_ and Ca, or pair feeding in thermoneutrality. (**a**) Heat map of the top 30 fecal metabolites influenced by treatment. (**b**–**r**) Treatment differences in rectal temperature at 08h00, 14h00, and 17h00 (rec. temp.), respiratory rate at 17h00 (resp. rate 17h00), milk somatic cells (milk SCC), plasma tumor necrosis factor–alpha (TNF-α), lipopolysacaride-binding protein (LBP), tridecylic acid, myristic acid, non-esterified fatty acids prior to feeding (NEFA pre-F), vitamin B_7_, histidine, p-hydroxyphenilacetic acid (p-HPHA), acetyl-ornitine, methylhistidine, arginine, and retinol identified in ANOVA with the false discovery rate (FDR) method. Normalized, pareto-scaled concentration data are representative of fecal metabolites collected from multiparous Holstein dairy cows (*n* = 12 observations per treatment). For visualization purposes, the heat map represents log-transformed, pareto-scaled data to showcase concentrations in a color gradient as high (red) or low (blue). Panels represent significant treatments differences with *P*_FDR_ < 0.05. Treatments were: (1) heat stress (HS); (2) heat stress with higher dietary concentrations of vitamin D_3_ and Ca (HS+DCa; 1820 IU/kg and 1.5% Ca); (3) thermoneutral, pair-feeding (TNPF). Treatments were allocated within two plots differing in basal concentrations of vitamin E and Se (high: 200 IU/kg vit. E and 1.2 mg/kg Se; low: 20 IU/kg vit. E and 0.3 mg/kg Se). Data are averaged across plots as no treatment × plot interactions were detected. Fecal metabolite data were obtained using a LC–MS/MS custom assay. Heatmap abbreviation: pref. FA yield = milk preformed fatty acid yield; insulin post-F = plasma insulin 4 h after feeding; CRP = plasma C-reactive protein.

The ANOVA (Table 1, Figure 2) revealed 17 metabolites affected by treatment (*P*_FDR_ < 0.05). Examination of the differential metabolites (Table 1) indicates involvement in pathways of bile synthesis; biosynthesis of fatty acids, vitamins, and amino acids; and biomarkers of heat stress and inflammation.

Relative to TNPF, HS increased inflammatory markers such as TNF-α (+3.55-fold, *P*_FDR_ < 0.001; Table 1), LBP (+1.74-fold, *P*_FDR_ < 0.001; Table 1), milk somatic cells (milk SC, + 4.10-fold, *P*_FDR_ < 0.001; Table 1), respiration rate (+ 2.48-fold, *P*_FDR_ < 0.001; Table 1), and rectal temperature at 08h00, 14h00, and 17h00 (+1.01-, +1.02-, and +1.06-fold, respectively, *P*_FDR_ < 0.05; Table 1). Amino acid biosynthesis was also affected by HS relative to TNPF, as fecal concentration of amino acids such as histidine, acetyl-ornithine, methyl histidine, and arginine were decreased in HS cows by −0.12-, −0.34-, −0.89-, and −0.42-fold (*P*_FDR_ < 0.001; Table 1), respectively. The fecal fatty acids tridecylic and myristic acids were increased (*P*_FDR_ < 0.001; Table 1) in HS cows relative to TNPF by +2.06- and +1.87-fold, respectively. Fecal concentration of the vitamins B_7_ and retinol increased (*P*_FDR_ < 0.001; Table 1) by +1.58- and +1.41-fold in HS conditions relative to TNPF, respectively. Lastly, concentrations of p-hydroxyphenylacetic acid (p-HPHA), a by-product of microbial fermentation, was found to be +2.44-fold higher in the feces of HS cows by relative to TNPF (*P*_FDR_ < 0.001; Table 1).

Relative to HS, HS+DCa decreased markers of heat stress and inflammation, such as rectal temperature at 17h00 by −1.02-fold (*P*_FDR_ < 0.001; Table 1), TNF- α by −2.08-fold (*P*_FDR_ < 0.001; Table 1), milk SCC by −2.31-fold (*P*_FDR_ = 0.031; Table 1), and LBP by −1.45-fold (*P*_FDR_ < 0.01; Table 1). In addition, HS + DCa increased fecal concentration of methyl histidine by +0.36-fold relative to HS cows (*P*_FDR_ < 0.01).

**Figure 3:**
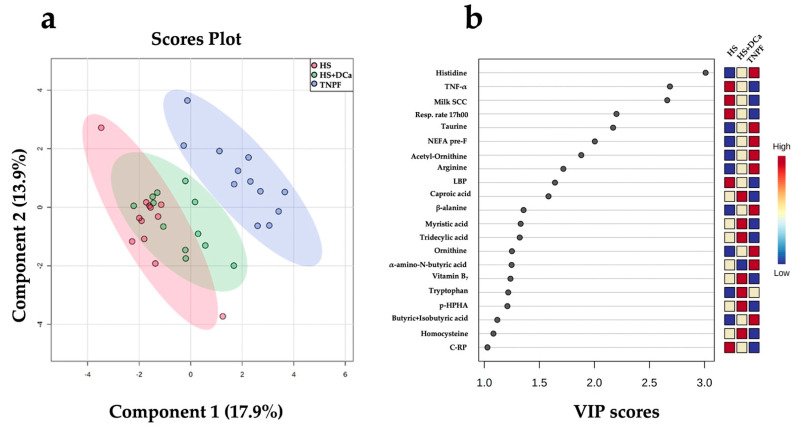
Discriminant analysis of fecal metabolites of dairy cows following heat stress (HS), heat stress with vitamin D_3_ and Ca (HS+DCa), or pair feeding in thermoneutrality (TNPF). (**a**) Two-dimensional partial least squares discriminant (PLS-DA) score plot. (**b**) Variable importance projection (VIP) scores analysis based on component 1 of the PLS-DA used to rank the relative contribution of metabolites to the variance between dietary treatments. Treatments were: (1) heat stress (HS); (2) heat stress with higher vitamin D_3_ and Ca (HS+DCa; 1820 IU/kg and 1.5% Ca); (3) thermoneutral, pair-feeding (TNPF). Fecal metabolites data were obtained using a LC–MS/MS custom assay. Fecal metabolite data were obtained using an LC–MS/MS custom assay. p-HPHA = plasma p-hydroxyphenilacetic acid; C-RP = plasma C-reactive protein.

Hierarchical clustering showed that fecal metabolites and physiological parameters could be grouped according to their differential enrichment by treatment group (Figure 3a), where myristic acid; tridecylic acid; milk somatic cells, rectal temperature (rect. temp.) at 08h00, 14h00, and 17h00; tumor necrosis factor–alpha (TNF-α); vitamins of B group (B_7_ and B_9_); lipopolysaccharide-binding protein (LBP); p-hydroxyphenylacetic acid (p-HPHA); and retinol concentrations were generally increased in both heat stress groups.

Partial least squares discriminant analysis (PLS-DA) revealed a distinct separation between the HS and TNPF groups (*p* = 0.001; Figure 3a). Cross-validation across two components with 999 random permutation tests yielded significant *p*-values that were found for Q_2_ = 0.522, *p* = 0.001, and R_2_Y = 0.816, *p* = 0.001, respectively. The variable importance projection (VIP) scores analysis on component 1 identified 21 variables with a VIP score > 1 (Figure 3b). Among these markers, respiratory rate at 17h00, TNF-α, LBP, milk SCC, and C-reactive protein (C-RP) were the highest in the HS group, whereas taurine, NEFA, acetyl-ornithine, arginine, β-alanine, ornithine, and tryptophan were the lowest relative to the other groups. On the other hand, caproic acid (6:0), tridecylic acid (13:0), myristic acid (14:0), tryptophan, p-HAA, and homocysteine concentrations were highest in the HS+DCa group in intermediate in HS, whereas only tryptophan provided a clear separation, exhibiting the highest concentrations in HS+DCa and the lowest in HS (Figure 3b).

### 2.3. Changes in Fecal Metabolites Related to the Level of Vitamin E and Selenium of Dairy Cows

**Figure 4:**
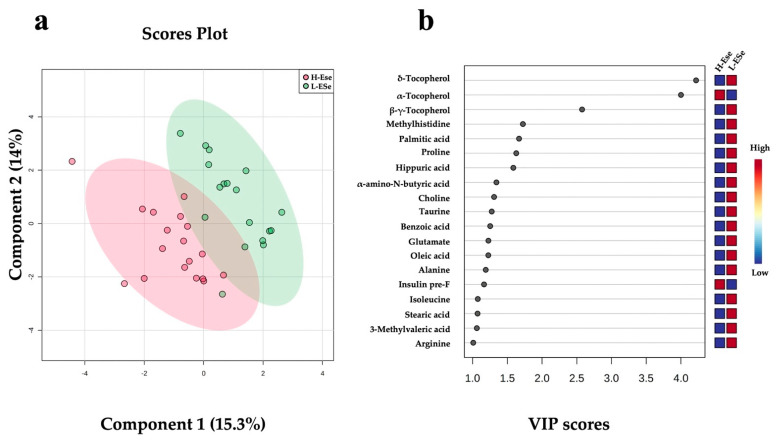
Discriminant analysis of fecal metabolites of dairy cows fed high vitamin E and Se (H-ESe) or low vitamin E and Se (L-ESe). (**a**) Two-dimensional partial least squares discriminant (PLS-DA) score plot. (**b**) Variable importance projection (VIP) scores analysis based on Component 1 of the PLS-DA used to rank the relative contribution of metabolites to the variance between dietary treatments. Treatments were two plots differing in basal concentrations of vitamin E and Se (high: 200 IU/kg vit. E and 1.2 mg/kg Se; low: 20 IU/kg vit. E and 0.3 mg/kg Se). Fecal metabolite data were obtained using a LC–MS/MS custom assay. Insulin pre-F = plasma insulin prior to feeding.

Partial least squares discriminant analysis (PLS-DA) plots of the fecal metabolomic data revealed unique features predictive of vitamin E and Se (H-ESe or L-ESe) feeding (*p* < 0.05; Figure 4a). The cross validation across two components with 999 random permutation tests was performed, and significant *p*-values were found for Q_2_ = 0.323, *p* = 0.02, and R_2_Y = 0.734, *p* = 0.03, respectively. A total of 19 fecal metabolites exhibited VIP scores > 1, including two different forms of tocopherol (∆, and β + γ), methylhistidine, palmitic acid, proline, hippuric acid, α-amino-*N*-butyric acid, choline, taurine, benzoic acid, glutamate, oleic acid, alanine, isoleucine, stearic acid, 3-methylvaleric acid, and arginine. These metabolites were all associated to the lower vitamin E and Se, whereas in the high level of supplementation, α-tocopherol and pre-prandial plasmatic insulin were associated with the higher vitamin E and Se (Figure 4b).

Three vitamin E metabolites were different among the H-ESe and L-ESe plots in the ANOVA (*P*_FDR_ < 0.05; Table 2). A greater concentration of α-tocopherol by +3.20-fold was observed in the H-ESe group (*P*_FDR_ < 0.05), while ∆-tocopherol and β + γ-tocopherol were reduced in H-ESe relative to L-ESe (−0.23- and −0.51-fold, respectively; *P*_FDR_ < 0.05).

### 2.4. Correlation of Selected Fecal Metabolites with Leaky Gut and Inflammation Markers, Lactation Performance and Clinical Parameters

Significant correlations were observed for many of the fecal metabolites and leaky gut markers (Table 3). Calprotectin, a proxy for leaky gut, correlated negatively (*p* < 0.01 or *p* < 0.001) with most of the B-group vitamins, amino acids (e.g., citrulline, glycine, histidine, tyrosine, valine, isoleucine, leucine, methionine, ornithine, phenylamine, serine, threonine, and tryptophan), choline, volatile fatty acids (acetic, butyric + isobutyric, and 2-methylbutyric acids), and fatty acids (heptanoic, caprylic, capric, undecylic, and lauric acids). Inflammatory markers, such as TNF-α and C-RP, correlated positively with vitamin B1 and B7, retinol, amino acids (arginine, asparagine, histidine, and acetyl-ornithine), acetic acid, and fatty acids (tridecylic acid and myristic acid; all *p* < 0.01). Lactation performance variables, such as DMI, milk yield, and the yields of milk components (i.e., fat, protein, and lactose), were negatively correlated with retinol, glutamine, leucine, 2-methylbutiric acid, capric acid, and myristic acid (Table 3, *p* < 0.01). Plasma insulin concentration correlated positively with retinol and negatively with fecal amino acids such as arginine, aspartate, glutamate, histidine, valine, ornithine, choline, acetyl-ornithine, and β-alanine, as well as with fecal VFA such as acetic, propionic, and butyric acids (Table 3, *p* < 0.01), whereas 3-methylvaleric acid, caproic acid, tridecylic acid, and oleic acid correlated positively with plasmatic insulin concentration. Furthermore, rectal temperatures, respiratory rates, and milk somatic cells correlated positively with all fecal metabolites. Similarly, these variables were positively correlated with inflammatory markers, fecal B-group vitamins, and fecal amino acids such as arginine, histidine, valine, ornithine, tryptophan, and acetyl-ornithine. Fecal taurine concentrations correlated negatively with rectal temperature and milk somatic cells (Table 3, *p* < 0.001).

## 3. Discussion

In the present study, changes in fecal metabolite profiles were evaluated at 14 days relative to the start of treatments using a Latin square design balanced for residual effects. This experimental design allowed us to account for variation related to individuals and experimental periods [24]. The length of experimental periods was based on previous work by Wheelock et al. [5], wherein a stable heat stress response was established within 5 days. Furthermore, the washout periods (7 days) were sufficient for the recovery of key response variables, such as dry matter intake, milk yield, rectal temperatures, inflammation, and permeability markers (data not shown). In addition, in order to prevent residual effects of vitamin E and Se supplementation, we randomly assigned animals to one of the blocks (i.e., L-ESe or H-ESe). Considering that feeding dynamics could alter the flux of intestinal metabolites and gut microbiota composition [25,26], we conceived our experimental design to control for the effect of feed intake levels, thus enabling us to evaluate thermal stress while eliminating the confounding effects of dissimilar nutrient intake between the TNPF and HS groups.

Although some fecal metabolites may originate from endogenous losses [22], others are the direct reflection of lower gut fermentation characteristics, which in turn are related to alterations of the gut microbiota composition [23]. Like the rumen, the large intestine of the cow hosts a diverse community of microorganisms, including bacteria, protozoa, and fungi [27]. Some of these microorganisms may contribute to changes in the fecal microbiota metabolome, as they possess cellulase, protease, deaminase, and urease activities and thus release fermentation products and other metabolites, including VFA, nitrogenous compounds, lipids, and vitamins [27,28].

In the present study, ≈98% of identified fecal metabolites corresponded to VFA (e.g., acetate, propionate, and butyrate), the main products of microbial fermentation of carbohydrates [29]. Although some diets (e.g., high-starch diets) can alter fecal VFA concentrations [29], the effects of heat stress remain poorly understood. However, limited available data shows reduced pH and increased lactate concentrations in rumen fluid of heat-stressed cows [30]. In the present experiment, individual VFA concentrations in feces were not affected by treatment, although they were negatively associated with heat stress indicators, such as rectal temperatures and respiratory rates, suggesting a negative impact of heat stress on lower gut fermentation. However, in contrast to a previous report, butyrate and not propionate was the second predominant VFA after acetate [31]. The reason for this discrepancy is not clear. Similar to our observations, total rumen VFA concentrations have also been shown to be reduced by heat stress [32]. In addition, Xiong et al. [33] observed that finishing pigs under heat stress presented lower fecal concentration of total short-chain fatty acids: acetic, propionic, butyric, valeric, and isovaleric acids; the previous study suggested that microbes slowed down fermentation activity in the lower gut.

The extent to which fecal metabolites are associated with changes in microbial communities was not investigated in the present study. However, some fecal metabolites identified herein could serve as proxies of changes in fecal microbiota composition and/or metabolism induced by diet and heat stress. Indeed, despite receiving the same ration and a displaying similar levels of feed intake, the HS and TNPF groups differed in the concentrations of some fecal metabolites such as histidine, p-HPHA, vitamin B_7_, tridecylic acid, myristic acid, arginine, and acetyl ornithine, suggesting alterations in microbial metabolic pathways. Indeed, heat stress increased fecal concentrations of p-HPHA (indicator of bacterial flavonoid metabolism [34]), as well as vitamin B_7_ (bacterial vitamin metabolism [35]) and 13:0 and 14:0 (bacterial lipid metabolism [36]). Metabolites related to bacterial synthesis of amino acids (i.e., histidine, acetyl-ornithine, and arginine) were lower in the feces of heat-stressed cows. Interestingly, acetyl-ornithine is a direct precursor of the bacterial arginine synthesis pathway [37], thus explaining their simultaneous reduction in the heat stress group.

There is precedent in the use of fecal metabolite profiles for the study of heat stress and its discriminating biomarkers in domestic animals. For instance, He et al. [38] reported significant alterations in fecal concentrations of several metabolites in gestating sows experiencing heat stress. In agreement with our observations, the authors reported negative reduced β-alanine and VFA in fecal samples of heat-stressed animals. In addition to traditional markers of heat stress (e.g., respiratory rates), plasma NEFA and inflammation (LBP, C-RP, and TNF-α), our analyses identified a number of fecal biomarkers that discriminate animals subjected to heat stress from their thermoneutral counterparts. Reduced concentrations of β-alanine, histidine, tryptophan, ornithine, acetyl ornithine, and arginine suggest that microbial amino acid metabolism may be a key feature predictive of the heat stress phenotype. In addition, lower concentrations of taurine in the HS group allowed for discrimination from TNPF cows.

Although not many changes were observed in response to vitamin D_3_ and Ca supplementation during heat stress, p-HPHA concentrations were significantly higher in HS+DCa than in the HS group, suggesting these nutrients may influence microbial metabolism [34]. Similarly, relative to HS, HS+DCa increased fecal methylhistidine, which has been proposed as a marker of muscle catabolism, inflammation, and heat stress [39,40]. These effects of vitamin D_3_ and Ca were observed, despite reductions in rectal temperatures, respiratory rates, and inflammation markers. We thus speculate that rather than originating from endogenous losses, methylhistidine in feces may be derived from alteration of intestinal microbial metabolism. In addition, tryptophan concentrations were higher in HS+DCa than in HS and discriminated between the two groups. Of note, tryptophan can indirectly reduce inflammation and improve gut barrier integrity via its transformation into tryptophan derivatives by gut microbiota [41]. Interestingly, in the present study, vitamin D_3_ and Ca supplementation during heat stress reduced rectal temperatures, respiratory rates, TNF-α, and LBP relative to heat stress, which were in general negatively associated with fecal tryptophan. Febrile response occurs directly as a reaction to endotoxemia [42], which is typically observed under heat stress. In line with this observation, HS cows in the present experiment likely experienced endotoxemia, as suggested by elevated LBP and TNF-α concentrations, characteristic of the pyrogenic cytokine response via Toll-like receptor 4 [42]. The reduction in body temperatures observed in the vitamin D-supplemented group are thus likely explained by a reduction in proinflammatory cytokines (e.g., TNF-α) [43]. Furthermore, tryptophan concentrations in feces presented a moderate inverse correlation (*r* = −0.63) with fecal calprotectin, which is an indirect marker of gut permeability, which was increased during HS in the present experiment. This is in keeping with the observation that fecal tryptophan concentrations can also discriminate between the HS (highest) and TNPF (lowest) groups. Together, these observations suggest that the partial alleviation of heat stress in the HS+DCa group may at least in part be explained by the stimulation of intestinal tryptophan synthesis.

Similar to tryptophan, the amino acid taurine was lower in HS relative to TNPF and discriminated between these groups. Although the direct effects of heat stress on taurine metabolism and intestinal secretion have not been extensively described, rodent data show that in responding to infection, animals can increase intestinal secretion of taurine, which microbiota could then use to produce sulfide and inhibit pathogen respiration [20]. In contrast, fecal taurine concentrations were lower in HS relative to TNPF cows, which suggests that increased intestinal taurine secretion is not a feature of the response to HS-related endotoxemia. Taurine plays an important antioxidant role, acting to protect immune cells from oxidative stress [44,45], a function that may be relevant during heat stress. Although the reason for reduced fecal taurine concentrations are unclear, we speculate that heat stress reduces taurine synthesis and/or secretion into the intestinal lumen, due to either reduced substrate availability or prioritization for immune cell protection from oxidative stress. A variety of antioxidants are involved in the prevention of oxidant-induced cell damage and reduction of oxidative modification of lipids, proteins, and DNA [46]. However, in the present study, when vitamin E and Se were supplemented to heat-stressed cows, only the fecal concentrations α- and δ-tocopherol were affected, while no impact on inflammatory gut integrity markers were observed.

## 4. Materials and Methods

### 4.1. Experimental Design and Treatments

Animal care condition and management practice were approved by the animal care committee (2019-BL-386) of the Centre de Recherche en Sciences Animales de Deschambault (CRSAD), following the regulations of the Canadian Council on Animal Care (1993) [47]. Twelve multiparous Holstein cows (42.2 ± 5.6 kg milk/d; 83.4 ± 27.1 days in milk) were used to test the effects of Vitamin D_3_ and Ca supplementation on systemic inflammation during HS under diets differing in basal concentrations of vitamin E and Se in a split-plot design. The main plot was vitamin E and Se supplementation level: (1) control (20 IU/kg vit. E and 0.3 mg/kg Se, according to NRC [48]; *n* = 6) or (2) high vitamin E, high Se (200 IU/kg vit. E and 1.2 mg/kg Se, i.e., 10- and 4-fold above dietary recommendations, respectively; *n* = 6). Within each main plot, cows were assigned to either heat stress (HS), pair feeding in thermo-neutrality (PFTN), or HS with vitamin D_3_ and Ca supplementation (HS+DCa, 1820 IU/kg, and 1.5% Ca) in a duplicated 3 × 3 Latin square with 14 day periods. Animals in the TNPF group were pair-fed with HS animals to eliminate the confounding effects of dissimilar nutrient intake. During the three experimental periods, the reduction in feed intake in PF cows was calculated on the basis of the intake reduction observed in the HS group. Experimental diets were based on corn and alfalfa silages, ground corn, and soya with a forage to concentrate ratio of 80:20, and only differed in the type of mineral premix according to treatment. Diets provided 26.7% NDF, 15.9% CP, 30.6% starch, and 2.50% fatty acids (DM-basis). All cows were individually fed a total mixed ration (TMR) two times daily (09h00 and 14h00), and refusals were recorded daily before the first day feeding. During all 3 experimental periods, HS cows were allowed to eat ad libitum. However, thermoneutral PF cows were limit-fed as described above.

### 4.2. Environmental and Physiological Parameter Measurements

Heat-stressed cows experienced cyclical daily temperatures (to mimic diurnal patterns), ranging from 29.0 to 39.0 °C with 20 to 50% of humidity (THI = 72.0−82.0). Cows in the thermoneutral PF group remained at 20 °C, with humidity ranging between 55 and 64% (THI = 61.0–64.0). All cows were kept under a 12 h light and dark cycle. The daily heat stress cyclical pattern was constant during the three experimental periods, taking into consideration characteristics of climate chambers, which have been previously reported [4,49]. In between experimental periods, experimental cows (HS and PFTN) switched chambers and were acclimated under thermo-neutral conditions (THI = 61.0–64.0, 12 h light and dark cycle) during 7 days as a recovery week. Rectal temperatures and respiration rates were measured at 08h00 (at lowest THI), 14h00, and 17h00 (at peak THI).

### 4.3. Sample Collection and Analysis

Fecal and pre-prandial blood samples were collected in the morning at 07h00, and post-prandial blood samples were collected 4h after feeding on day 14 of each period. Blood samples were centrifuged at 1500 × *g* at 4 °C for 15 min, and then the plasma was collected and placed into 2 mL Eppendorf tubes. Fecal and plasma samples were frozen at −20 °C until analyzed. Fecal samples were analyzed for targeted quantitative LC–MS and for calprotectin concentration. Plasma samples were analyzed for lipopolysaccharide binding protein (LBP), C-reactive protein (C-RP), and tumor necrosis factor-alpha (TNF-α) concentrations. Fecal calprotectin and LBP, C-RP, and TNF-α were assessed using commercial immunoassay kits (MyBiosource, San Diego, CA, USA). Plasma insulin concentration was determined by immunoassay (Mercodia, Uppsala, Sweden). Plasma NEFA concentration was determined using an enzyme-based colorimetric assay (Wako HR series NEFAHR-2 kit; Wako Chemicals USA Inc., Richmond, VA, USA). Cows were milked twice daily at 07h30 and 16h30, and milk yield was determined by an integrated milk meter (Flomaster Pro; DeLaval, Tumba, Sweden) on day 14 in each period. At both milking, two samples of milk per cows were collected in tubes with preservative Bronopol (2-bromo-2-nitropropane-1,3-dio) and stored at 4 °C.

#### 4.3.1. Targeted Quantitative Metabolomics

Targeted quantitative metabolomics were performed using reverse-phase liquid chromatography and tandem mass spectrometry, using and Agilent 1260 series UHPLC system (Agilent Technologies, Palo Alto, CA, USA) coupled with an ABSciex 4000 Qtrap^®®^ tandem mass spectrometry (AB Sciex, Concord, ON, Canada). The method combines the derivatization and extraction of analytes, and the selective mass-spectrometric detection using multiple reaction monitoring (MRM) pairs. Isotope-labeled internal standards and other internal standards were used for metabolite quantification.

For most metabolites, except for organic acids, samples were thawed on ice, vortexed, and centrifuged at 13,000× *g*. Samples (10 µL) were dried under N_2_ and derivatized with phenyl-isothiocyanate. Metabolites were extracted with 300 µL of extraction solvent.

For organic acids, 150 µL of ice-cold methanol and 10 µL of isotope-labeled internal standard mixture were added to 50 µL of sample for overnight protein precipitation and then centrifuged at 13,000× *g* for 20 min. Supernatants (50 µL) were obtained, and 3-nitrophenylhydrazine (NPH) reagent was added. Following a 2h incubation, BHT stabilizer and water were added prior to LC–MS injection. Data analysis was conducted using the Sciex Analyst 1.6.2 (AB Sciex, Concord, ON, Canada).

#### 4.3.2. Vitamin Analysis

Water-soluble and fat-soluble vitamins were analyzed a targeted analysis method using previously described methods [50]. To quantify the water-soluble vitamins, we generated 7-point calibration curves by adding 10 μL of the isotopically labeled internal standard mixture to 50 μL of the calibration solutions. Similarly, fecal extracts were prepared by adding the isotopically labeled internal standard mixture to 50 μL of sample. A total of 60 μL of an aqueous TCA solution (50 mg/mL) was added to tubes containing calibrants or samples. Each tube was vortexed for 30 s for thorough mixing and then left on ice for 1 h. After cooling, samples were centrifuged at 13,000 rpm for 20 min, and 100 μL of the supernatants was collected. A volume of 10 μL was injected for LC−MS/MS analysis using the above-describe system. An Agilent reversed-phase Zorbax Eclipse XDB C18 column (3.0 mm × 100 mm, 3.5 μm particle size, 80 Å pore size) coupled to a Phenomenex (Torrance, CA, USA) SecurityGuard C18 precolumn (4.0 mm × 3.0 mm) was used for the separation of all water-soluble vitamins in the samples. For fat-soluble vitamin analysis, 50 μL of calibration solution or samples were mixed with 50 μL of internal standard mixture solution. Subsequently, a methanol and 0.2 M ZnSO_4_ mixture solution (300 μL, 1:1 *v/v*) was added to precipitate the proteins and to facilitate the release of 25-hydroxyvitamin D_3_ from vitamin D-binding protein. Following precipitation, 1 mL of hexane was added to every sample to extract the fat-soluble vitamins. Samples were vortexed for 10 min and centrifuged at 13,000 rpm for 20 min. The hexane layer (650 μL) was then subjected to evaporation under N_2_ gas at 40 °C until dried. Finally, 200 μL of methanol was added to each dried sample to reconstitute the analytes, and 10 μL was injected for LC−MS/MS analysis, as described above. A Phenomenex Kinetex C18 column (3.0 mm × 100 mm, 2.6 μm particle size, 100 Å pore size) connected to a Phenomenex SecurityGuard C18 precolumn (4.0 mm × 3.0 mm) was used to separate the fat-soluble vitamins. Data analysis was conducted using the Sciex Analyst software 1.6.2 (AB Sciex, Concord, ON, Canada).

#### 4.3.3. Milk Composition Analyses

Milk samples were analyzed for fat, protein, lactose, and β-hydroxybutyrate (BHBA; Lactanet, Sainte-Anne-de-Bellevue, QC, Canada), as well as for the analysis of the milk components by IR absorption spectroscopy with a Foss MilkoScan FT 6000 instrument (Foss, Hillerød, Denmark). The same sample was used for somatic cell counting using a Fossomatic FC (Foss, Fossomatic technology, Hilleroed, Denmark).

### 4.4. Multivariate Data Analysis

We analyzed lipidomic data using the web server MetaboAnalyst 5.0 (www.metaboanalyst.ca; accessed on 22–24 November 2021 [51]). Non-filtered data were normalized by the sum method, generalized log-transformed, and Pareto-scaled. Multivariate analysis of data included partial least squares discriminant analysis (PLS-DA), ANOVA, and Pearson’s correlation coefficient procedures. Significance was declared at *p* ≤ 0.05 and false discovery rate (FDR) at <0.05. For visualization purposes, heat maps were generated using generalized log-transformed, normalized, and pareto-scaled data to showcase the magnitude of fold-change in a color gradient for increased (red) or decreased (blue) relative abundance.

## 5. Conclusions

Heat stress and diet altered the fecal metabolome of dairy cows. In conclusion, both heat stress and the supplementation of vitamin D_3_ and Ca can influence the fecal metabolome of dairy cows independently of dietary levels of vitamin E and Se. Our results suggest that some fecal metabolites are well associated with physiological measures of heat stress, inflammation, and leaky gut, thus potentially providing insights into the gut-level changes taking place in dairy cows under these conditions. Because the microbiome is a major contributor to the metabolite composition of feces, future studies should investigate how these metabolites associate with microbiota during periods of heat stress.

## Figures and Tables

**Table 1:** Physiological parameters and fecal metabolites affected by treatment * in ANOVA, as identified by LC–MS/MS.

Item	Metabolic Pathway	FC HS vs. TNPF ^a^	FC HS vs. HS+DCa ^b^	ANOVA	*P*_FDR_ HS vs. TNPF ^d^	*P*_FDR_ HS vs. HS+Dca ^e^
				*P*_FDR_^c^		
Rectal temperature 17h00 TNF-α	Heat stress Inflammation	1.06 3.55	−1.02 −2.08	<0.001 <0.001	<0.001	<0.001
					<0.001	0.02
Respiratory rate 17h	Heat stress	2.48	−1.15	<0.001	<0.001	ns
Milk SCC	Inflammation	4.1	−2.31	<0.001	<0.001	0.03
Rectal temperature 14h00	Heat stress	1.02	−0.99	<0.001	<0.001	ns
LBP	Heat stress	1.74	−1.45	<0.001	<0.001	0.006
Tridecylic acid	Microbial metabolism	2.06	0.71	<0.001	0.03	ns
Myristic acid	Fatty acid metabolism	1.87	0.79	<0.001	0.02	ns
NEFA pre-F	Fatty acid metabolism	−0.92	1.01	<0.001	<0.001	ns
Vitamin B_7_	Vitamin metabolism	1.58	0.9	<0.001	<0.001	ns
Histidine	Amino acid synthesis	−0.12	0.42	<0.001	<0.01	ns
p-HPHA	Microbial metabolism	2.44	0.43	<0.001	<0.01	<0.01
Acetyl ornithine	Amino acid synthesis	−0.34	0.91	<0.001	0.01	ns
Methyl histidine	Amino acid synthesis	−0.89	0.36	0.02	ns	0.01
Arginine	Amino acid synthesis	−0.42	0.71	0.02	0.02	ns
Rectal temperature 08h00	Heat stress	1.01	−1.00	0.02	0.04	ns
Retinol	Metabolism of vitamins	1.41	−0.92	0.04	ns	ns

* Treatments were: (1) heat stress (HS); (2) heat stress with higher dietary concentrations of vitamin D_3_ and Ca (HS+DCa; 1820 IU/kg and 1.5% Ca); (3) thermoneutral, pair-feeding (TNPF). Treatments were allocated within two plots differing in basal concentrations of vitamin E and Se (high: 200 IU/kg vit. E and 1.2 mg/kg Se; low: 20 IU/kg vit. E and 0.3 mg/kg Se). Data are averaged across plots, as no treatment × plot interactions were detected. ^a^ FC, fold change in the metabolite concentration (HS/TNPF). ^b^ FC, fold change in the metabolite concentration (HS/ HS+DCa). ^c^ *P*_FRD_, *p*-value of the false discovery rate by ANOVA analysis of fecal metabolites and variables of interest for HS, HS+ Ca, and TNPF. ^d^ *P*_FDR_ HS vs. TNPF, *p*-value of the false discovery rate by independent *t*-test for the preplanned contrast HS versus TNPF. ^e^ *P*_FDR_ HS vs. HS+DCa, *p*-value of the false discovery rate by independent *t*-test for the preplanned contrast HS versus HS+DCa. TNF-α = plasma tumor necrosis factor-alpha; LBP = plasma lipopolysaccharide-binding protein; p-HPHA = plasma p-hydroxyindoleacetic acid; NEFA pre-F = plasma non-esterified fatty acids prior to feeding.

**Table 2:** Fecal metabolites affected by treatment plot * in ANOVA, as identified by LC–MS/MS.

Fecal Metabolite	FC ^a^	log_2_(FC)	*p*-Value ^b^
α-Tocopherol	+3.20	1.67	<0.001
δ-Tocopherol	−0.23	−2.12	0.011
β + γ-Tocopherol	−0.51	−0.96	0.011

* Treatments were two plots differing in basal concentrations of vitamin E and Se (high: 200 IU/kg vit. E and 1.2 mg/kg Se; low: 20 IU/kg vit. E and 0.3 mg/kg Se). ^a^ FC, fold change in the metabolite concentration (H-ESe/L-ESe). ^b^ *p*-value, independent *t*-test for H-ESe versus L-ESe cows (*P*_FDR_ < 0.05).

**Table 3:** Partial Pearson’s correlation between fecal metabolite concentrations and indicators of inflammation, heat stress, metabolism, and performance across treatments *.

	Response Variable																
Metabolite	LBP	Calp	TNF	C−RP	DMI	MY	MFY	MPY	MLY	dNFA	mFA	pFA	NEFA	Ins	RT	RR	MSCC
Vitamin B_1_	−0.19	−0.51 *	−0.16	0.40 *	−0.06	−0.10	−0.14	−0.10	−0.09	−0.16	−0.05	−0.18	−0.18	0.04	−0.03	−0.13	−0.12
Vitamin B_2_	−0.27	−0.41 *	−0.31	−0.20	0.10	0.09	0.05	0.09	0.08	0.05	0.06	0.02	0.13	−0.28	−0.36	−0.51 *	−0.29
Vitamin B_5_	−0.27	−0.51 *	−0.22	−0.06	−0.04	−0.01	0.06	0.03	−0.02	0.03	0.09	0.06	0.00	−0.39	−0.27	−0.40 *	−0.29 *
Vitamin B_7_	0.55 **	0.10	0.41 *	0.56 *	−0.19	−0.09	−0.03	−0.04	−0.08	−0.01	−0.03	−0.07	−0.16	0.27	0.61 **	0.66 **	0.41
Vitamin B_9_	0.42 *	0.40 *	0.11	−0.29	0.19	0.12	−0.02	0.09	0.15	0.06	0.01	−0.13	−0.07	0.16	0.27	0.48 *	0.28
Retinol	0.24	0.20	0.49 *	0.43 *	−0.52 **	−0.56 *	−0.55 **	−0.62 **	−0.56 **	−0.56 **	−0.47 *	−0.55 *	−0.35	0.49 *	0.37 *	0.37	0.35
α-Tocopherol	0.02	0.08	−0.24	−0.19	−0.02	0.13	0.26	0.19	0.11	0.25	0.15	0.35	0.31	0.09	−0.30	−0.27	−0.33
β+γ-Tocopherol	−0.01	0.05	−0.30	0.02	0.09	0.21	0.14	0.19	0.22	0.13	0.12	0.20	0.38	−0.10	−0.01	0.14	−0.23
δ-Tocopherol	−0.23	0.10	0.00	−0.09	0.19	0.06	−0.02	0.05	0.07	−0.03	0.06	−0.08	−0.10	−0.43	0.08	−0.07	−0.09
Alanine	−0.23	−0.37	−0.16	−0.03	−0.25	−0.23	−0.10	−0.18	−0.22	−0.12	−0.08	−0.09	−0.10	−0.35	−0.18	−0.20	−0.35
Arginine	−0.51 *	−0.24	−0.46 *	−0.33	0.18	0.11	0.09	0.10	0.10	0.08	0.15	0.02	0.10	−0.52	−0.48 *	−0.64 **	−0.50 *
Asparagine	0.04	−0.16	0.31	0.41 *	−0.31	−0.32	−0.07	−0.20	−0.31	−0.12	−0.06	−0.01	−0.26	−0.05	0.27	0.17	0.00
Aspartate	−0.19	−0.06	0.02	0.12	0.05	−0.08	−0.08	−0.07	−0.10	−0.09	−0.05	−0.10	−0.11	−0.37 *	−0.05	−0.26	−0.13
Citrulline	−0.10	−0.46 *	0.22	0.31	−0.23	−0.34	−0.07	−0.26	−0.33	−0.12	−0.02	−0.08	−0.34	−0.14	0.09	−0.02	−0.04
Glutamate	−0.32	−0.32	−0.15	−0.03	−0.08	−0.11	0.02	−0.08	−0.12	−0.02	0.07	0.00	−0.08	−0.42 *	−0.17	−0.34	−0.33
Glutamine	0.04	−0.23	0.31	0.17	−0.51 *	−0.56 *	−0.34	−0.49 *	−0.57 **	−0.39 *	−0.34	−0.24	−0.26	0.04	0.11	0.06	0.07
Glycine	−0.15	−0.41 *	−0.08	0.14	−0.24	−0.18	−0.06	−0.14	−0.18	−0.10	0.01	−0.08	−0.12	−0.28	−0.07	−0.18	−0.24
Histidine	−0.55 **	−0.36 *	−0.51 *	−0.29	0.02	0.07	0.07	0.07	0.05	0.04	0.06	0.11	0.28	−0.47 *	−0.61 **	−0.73 **	−0.63 **
Tyrosine	−0.21	−0.33 *	0.06	0.21	−0.26	−0.33	−0.15	−0.27	−0.34	−0.18	−0.09	−0.19	−0.24	−0.21	−0.04	−0.24	−0.16
Valine	−0.45 *	−0.51 *	−0.16	0.12	−0.31	−0.24	−0.09	−0.19	−0.25	−0.16	−0.09	−0.01	0.01	−0.36 *	−0.27	−0.43 *	−0.43 *
Isoleucine	−0.29	−0.43 *	0.00	0.19	−0.35	−0.34	−0.16	−0.28	−0.35	−0.20	−0.13	−0.13	−0.16	−0.28	−0.11	−0.26	−0.27
Leucine	−0.31	−0.48 *	0.08	0.22	−0.41 *	−0.46 *	−0.27	−0.42 *	−0.46 *	−0.32	−0.22	−0.25	−0.23	−0.20	−0.09	−0.23	−0.19
Lysine	−0.26	−0.36	−0.15	0.04	−0.26	−0.21	−0.09	−0.17	−0.21	−0.13	−0.06	−0.07	−0.05	−0.35	−0.20	−0.26	−0.34
Methionine	−0.33	−0.46 *	−0.11	0.09	−0.23	−0.21	−0.02	−0.14	−0.22	−0.08	0.00	0.01	−0.13	−0.36	−0.23	−0.40 *	−0.31
Ornithine	−0.33	−0.51 *	−0.23	−0.04	−0.21	−0.15	−0.03	−0.09	−0.15	−0.07	−0.05	0.04	0.01	−0.38 *	−0.30	−0.35 *	−0.51 *
Phenylalanine	−0.29	−0.45 *	0.03	0.16	−0.29	−0.35	−0.16	−0.29	−0.36	−0.20	−0.11	−0.17	−0.23	−0.21	−0.11	−0.30	−0.21
Proline	−0.12	−0.35	0.01	0.21	−0.29	−0.23	−0.04	−0.15	−0.23	−0.10	−0.01	0.00	−0.17	−0.24	0.03	−0.06	−0.14
Serine	−0.13	−0.47 *	0.07	0.20	−0.34	−0.37	−0.17	−0.30	−0.37	−0.20	−0.12	−0.19	−0.27	−0.14	−0.03	−0.11	−0.13
Threonine	−0.29	−0.53 *	−0.07	0.11	−0.28	−0.29	−0.10	−0.22	−0.30	−0.17	−0.08	−0.06	−0.10	−0.35	−0.18	−0.33	−0.29
Tryptophan	−0.37	−0.63 **	−0.27	0.16	−0.25	−0.19	−0.08	−0.13	−0.20	−0.13	−0.04	−0.08	−0.05	−0.21	−0.28	−0.41 *	−0.40 *
Methylhistidine	−0.17	0.10	0.17	−0.05	0.05	−0.13	−0.04	−0.17	−0.15	−0.02	0.07	−0.19	−0.34	−0.24	0.02	−0.15	0.12
Choline	−0.30	−0.45 *	−0.20	−0.12	−0.12	−0.16	−0.08	−0.11	−0.15	−0.13	−0.06	−0.04	0.05	−0.45 *	−0.23	−0.20	−0.33
Acetyl-ornithine	−0.47 *	−0.30	−0.55 **	−0.24	−0.05	0.15	0.14	0.17	0.15	0.13	0.12	0.18	0.21	−0.47 *	−0.54 **	−0.52 **	−0.62 **
Taurine	−0.35	−0.23	−0.23	−0.19	−0.09	−0.16	−0.12	−0.16	−0.18	−0.17	−0.08	−0.11	0.00	−0.19	−0.33	−0.36 *	−0.32 *
Homocysteine	0.11	−0.03	0.26	0.04	−0.06	−0.18	−0.21	−0.17	−0.15	−0.22	−0.14	−0.26	−0.18	−0.18	0.39 *	0.30	0.14
trans-OH-Proline	0.04	−0.14	−0.23	0.17	0.10	0.26	0.37	0.31	0.26	0.31	0.32	0.43 *	0.17	0.01	−0.16	−0.14	−0.11
β-Alanine	−0.49	−0.16	−0.32	−0.10	0.16	0.12	0.03	0.09	0.10	0.03	0.03	0.04	0.21	−0.54 *	−0.27	−0.53 *	−0.55 *
Proline betaine	0.07	0.30	0.02	0.12	−0.04	0.01	0.09	0.04	0.02	0.14	0.15	−0.05	−0.03	−0.11	0.17	0.18	−0.02
α-Amino-N-butyric ac	−0.42	−0.23	−0.33	−0.03	−0.17	−0.04	−0.01	−0.04	−0.02	−0.01	−0.03	0.02	−0.02	−0.32	−0.30	−0.23	−0.44 *
γ-Aminobutyric	0.04	−0.38	−0.15	0.07	−0.11	0.19	0.28	0.25	0.20	0.22	0.23	0.37	0.14	−0.12	−0.08	0.05	−0.17
Acetic acid	−0.42 *	−0.42 *	−0.42 *	−0.15	0.08	0.14	0.22	0.16	0.14	0.21	0.24	0.20	0.09	−0.50 *	−0.45 *	−0.57 **	−0.57 **
Propionic acid	−0.26	−0.51 *	−0.29	0.03	0.02	0.11	0.23	0.14	0.11	0.21	0.22	0.24	0.04	−0.38 *	−0.30	−0.37 *	−0.43 *
Butyric+isobutyric acid	−0.41 *	−0.40 *	−0.34	−0.10	−0.08	0.08	0.19	0.09	0.07	0.13	0.21	0.21	0.12	−0.39 *	−0.43 *	−0.53 *	−0.41 *
2-Methylbutyric acid	−0.02	−0.40 *	0.05	0.14	−0.59 *	−0.44 *	−0.41 *	−0.43	−0.43 *	−0.41 *	−0.40 *	−0.34	0.05	0.12	−0.01	0.01	−0.14
Valeric+Isovaleric acid	−0.11	−0.22	0.04	0.17	−0.01	0.10	0.28	0.14	0.11	0.27	0.34	0.16	−0.19	−0.30	0.02	−0.11	0.01
3-Methylvaleric acid	0.03	0.21	0.17	−0.20	−0.05	−0.23	−0.28	−0.26	−0.23	−0.26	−0.22	−0.34	−0.13	0.38 *	0.08	0.19	0.07
Caproic acid	0.67 *	0.01	0.13	0.07	−0.04	−0.08	−0.18	−0.14	−0.07	−0.11	−0.19	−0.21	−0.03	0.59 *	0.27	0.50 *	0.34 *
Heptanoic acid	−0.05	−0.53 **	0.00	0.07	−0.36	−0.07	0.04	−0.03	−0.05	0.00	0.01	0.11	−0.01	−0.21	−0.03	0.02	−0.06
Caprylic acid	−0.06	−0.76 **	0.18	0.33	−0.34	−0.38	−0.30	−0.34	−0.35	−0.33	−0.26	−0.29	−0.30	0.06	0.13	0.09	0.03
Pelargonic acid	0.00	−0.31	−0.04	0.36	−0.45 *	−0.13	−0.01	−0.09	−0.11	−0.08	−0.04	0.09	0.06	0.14	0.06	0.21	0.08
Capric acid	−0.10	−0.68 **	0.29	0.33	−0.40 *	−0.44 *	−0.34	−0.42	−0.42 *	−0.39 *	−0.29	−0.30	−0.31	0.05	0.16	0.04	0.12
Undecylic acid	0.02	−0.68 **	0.26	0.31	−0.24	−0.26	−0.13	−0.21	−0.25	−0.19	−0.13	−0.05	−0.28	0.11	0.14	0.07	0.12
Lauric acid	−0.07	−0.73 **	0.12	0.26	−0.19	−0.18	−0.09	−0.14	−0.17	−0.17	−0.08	0.01	−0.18	0.10	0.01	−0.08	−0.05
Tridecylic acid	0.34	−0.07	0.38 *	0.52 *	−0.33	−0.24	−0.26	−0.21	−0.23	−0.27	−0.31	−0.17	−0.08	0.37 *	0.51 *	0.59 *	0.30 *
Myristic acid	0.34	−0.04	0.43 *	0.43 *	−0.34	−0.36	−0.45 *	−0.35	−0.35	−0.42 *	−0.46 *	−0.40 *	−0.12	0.28	0.55 **	0.61 *	0.30 *
Palmitic acid	−0.15	0.28	−0.24	−0.10	−0.03	−0.01	0.02	0.00	0.01	0.02	−0.05	0.09	0.25	0.14	−0.19	0.19	−0.14
Stearic acid	0.22	0.40	−0.08	−0.14	0.50 *	0.39	0.16	0.33	0.38	0.21	0.12	0.15	0.12	−0.06	0.12	0.21	0.13
Oleic acid	−0.08	0.42	−0.21	−0.15	0.35	0.37	0.27	0.33	0.36	0.33	0.17	0.29	0.20	−0.42 *	−0.10	−0.06	−0.10
Hippuric acid	0.02	0.27	−0.02	0.07	0.25	0.03	−0.07	−0.05	0.02	0.00	−0.10	−0.11	0.09	0.03	0.06	0.17	0.03
Benzoic acid	0.11	−0.09	0.29	0.27	−0.16	−0.11	0.05	−0.08	−0.10	0.00	0.09	0.03	−0.10	−0.02	0.30	0.23	0.31 *
^r^ p-HPHA	0.36	−0.06	0.32	0.27	−0.45 *	−0.29	−0.37	−0.34	−0.28	−0.34	−0.31	−0.43 *	−0.14	0.27	0.39 *	0.38 *	0.24

*p*-value of the significance * *p* < 0.01 or highly significant ** *p* < 0.001. Feature: LBP = plasma liposaccharide binding protein, Calp = fecal calprotectin, TNF-α = tumor necrosis factor-alpha, C-RP = C-reactive protein, DMI = dry matter intake, MY = milk yield, MFY = milk fat yield, MPY = milk protein yield, MLY = milk lactose yield, dNFA = milk de novo fatty acid yield, mFA = milk mixed-origin fatty acid yield, pFA = milk preformed fatty acid yield, NEFA = plasma non-esterified fatty acid concentrations, Ins = plasma insulin concentrations, RT = rectal temperature, RR = respiration rate, MSCC = milk somatic cell count, pHPHA = p-hydroxyphenylacetic acid, ac = acid. * Treatments were: (1) heat stress (HS); (2) heat stress with high vitamin D_3_ and Ca (HS+DCa; 1820 IU/kg and 1.5% Ca); (3) thermoneutral, pair-feeding (TNPF). Treatments were allocated within two plots differing in basal concentrations of vitamin E and Se (high: 200 IU/kg vit. E and 1.2 mg/kg Se; low: 20 IU/kg vit. E and 0.3 mg/kg Se). Data are averaged across plots as no treatment × plot interactions were detected. Fecal metabolite data were obtained using a LC–MS/MS custom assay.

## Data Availability

Data are contained within the article or Supplementary Material.

## Supplementary Materials

The following are available online at https://www.mdpi.com/article/10.3390/metabo12020142/s1, Figure S1: Concentrations of predominant fecal vitamins and choline (nmol/g of fresh feces) in dairy cows across treatments. Treatments were: 1-Heat stress; 2-Heat stress with higher Vitamin D and Ca (1820 IU/kg and 1.5% Ca); 3-Thermoneutral, pair-feeding. Treatments were allocated within two plots differing in basal concentrations of Vitamin E and Se (High: 200 IU/kg Vit. E and 1.2 mg/kg Se; Low: 20 IU/kg Vit. E and 0.3 mg/kg Se). Fecal metabolite data were obtained at day 14 of treatment initiation, and analyzed using a LC-MS/MS custom assay, Figure S2: Concentrations of amino acids (nmol/g of fresh feces) in dairy cows across treatments. (a) Essential amino acids, (b) Non-essential amino acids and (c) Other amino acids. Treatments were: 1-Heat stress; 2-Heat stress with higher Vitamin D_3_ and Ca (1820 IU/kg and 1.5% Ca); 3-Thermoneutral, pair-feeding. Treatments were allocated within two plots differing in basal concentrations of Vitamin E and Se (High: 200 IU/kg Vit. E and 1.2 mg/kg Se; Low: 20 IU/kg Vit. E and 0.3 mg/kg Se). Fecal metabolite data were obtained at day 14 of treatment initiation, and analyzed using a LC-MS/MS custom assay, Figure S3: Concentrations of fatty acids (nmol/g of fresh feces) in dairy cows across treatments. (a) short-chain fatty acids (<6C), (b) Medium chain fatty acids (6C–15C), (c) Long chain fatty acids (>16C). Treatments were: 1-Heat stress; 2-Heat stress with higher Vitamin D_3_ and Ca (1820 IU/kg and 1.5% Ca); 3-Thermoneutral, pair-feeding. Treatments were allocated within two plots differing in basal concentrations of Vitamin E and Se (High: 200 IU/kg Vit. E and 1.2 mg/kg Se; Low: 20 IU/kg Vit. E and 0.3 mg/kg Se). Fecal metabolite data were obtained at day 14 of treatment initiation, and analyzed using a LC-MS/MS custom assay, Figure S4: Concentrations of various fecal metabolites (nmol/g of fresh feces) in dairy cows across treatments. Fecal metabolites concentration was obtained using a LC-MS/MS custom assay. 3-(3-hydroxyphenyl)-3-hydroxypropionic acid (HPHPA), hippuric acid (HA), 5-Hydroxyindoleacetic acid (5-HIAA), benzoic acid (BZA), p-Hydroxyindoleacetic acid (p-HPHA), and salilylic acid (SA). Treatments were: 1-Heat stress; 2-Heat stress with higher Vitamin D_3_ and Ca (1820 IU/kg and 1.5% Ca); 3-Thermoneutral, pair-feeding. Treatments were allocated within two plots differing in basal concentrations of Vitamin E and Se (High: 200 IU/kg Vit. E and 1.2 mg/kg Se; Low: 20 IU/kg Vit. E and 0.3 mg/kg Se). Fecal metabolite data were obtained at day 14 of treatment initiation, and analyzed using a LC-MS/MS custom assay, Table S1: Metabolites concentration (nmol/g of fresh feces) in fecal samples of dairy cows using a LC-MS/MS custom assay. ND = not detected.
