# Supplementary material for: Modulation of Fecal Metabolites by Heat Stress and Diet, and Their Association with Inflammation and Leaky Gut Markers in Dairy Cows
Source: Metabolites. 2022 Feb 3;12(2):142. doi: 10.3390/metabo12020142 (PMC8874496; doi:10.3390/metabo12020142)
Supplement: Supplementary file 1 [file metabolites-12-00142-s001.zip › metabolites-1551820-supplementary.pdf]

metabolites

MDPI

Supplementary Materials

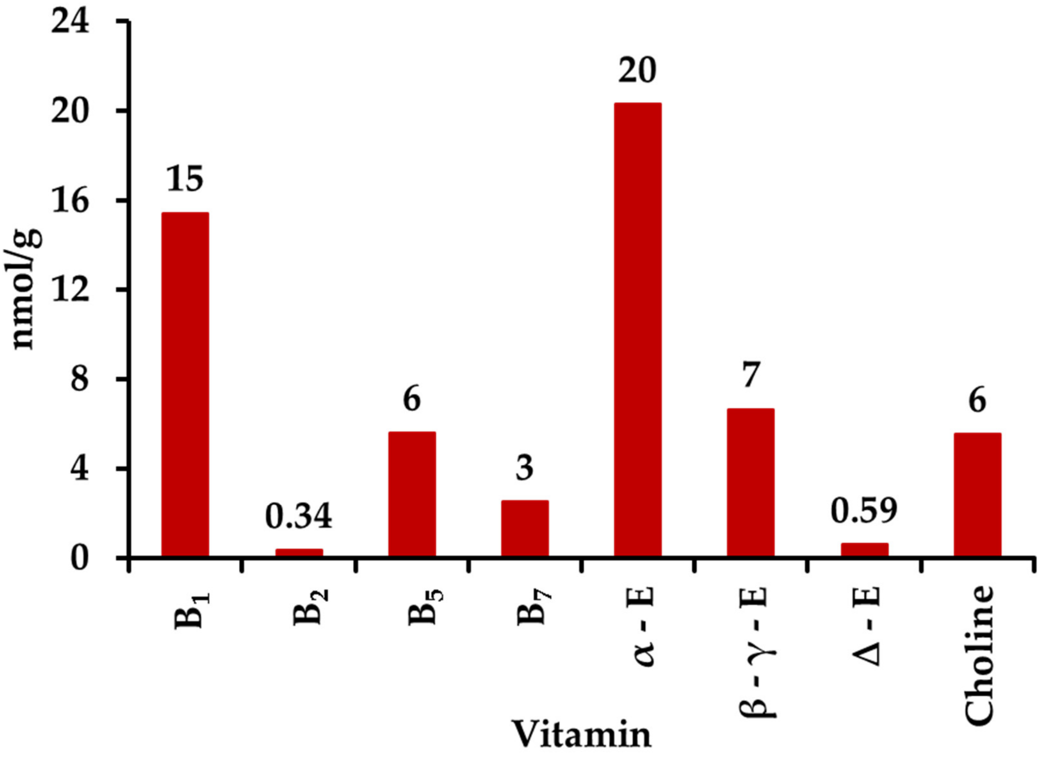

Supplementary Figure S1. Concentrations of predominant fecal vitamins and choline (nmol/g of fresh feces) in dairy cows across treatments. Treatments were: 1- Heat stress; 2- Heat stress with higher Vitamin D and Ca (1,820 IU/kg and 1.5 % Ca); 3- Thermoneutral, pair-feeding. Treatments were allocated within two plots differing in basal concentrations of Vitamin E and Se (High: 200 IU/kg Vit. E and 1.2 mg/kg Se; Low: 20 IU/kg Vit. E and 0.3 mg/kg Se). Fecal metabolite data were obtained at day 14 of treatment initiation, and analyzed using a LC-MS/MS custom assay.

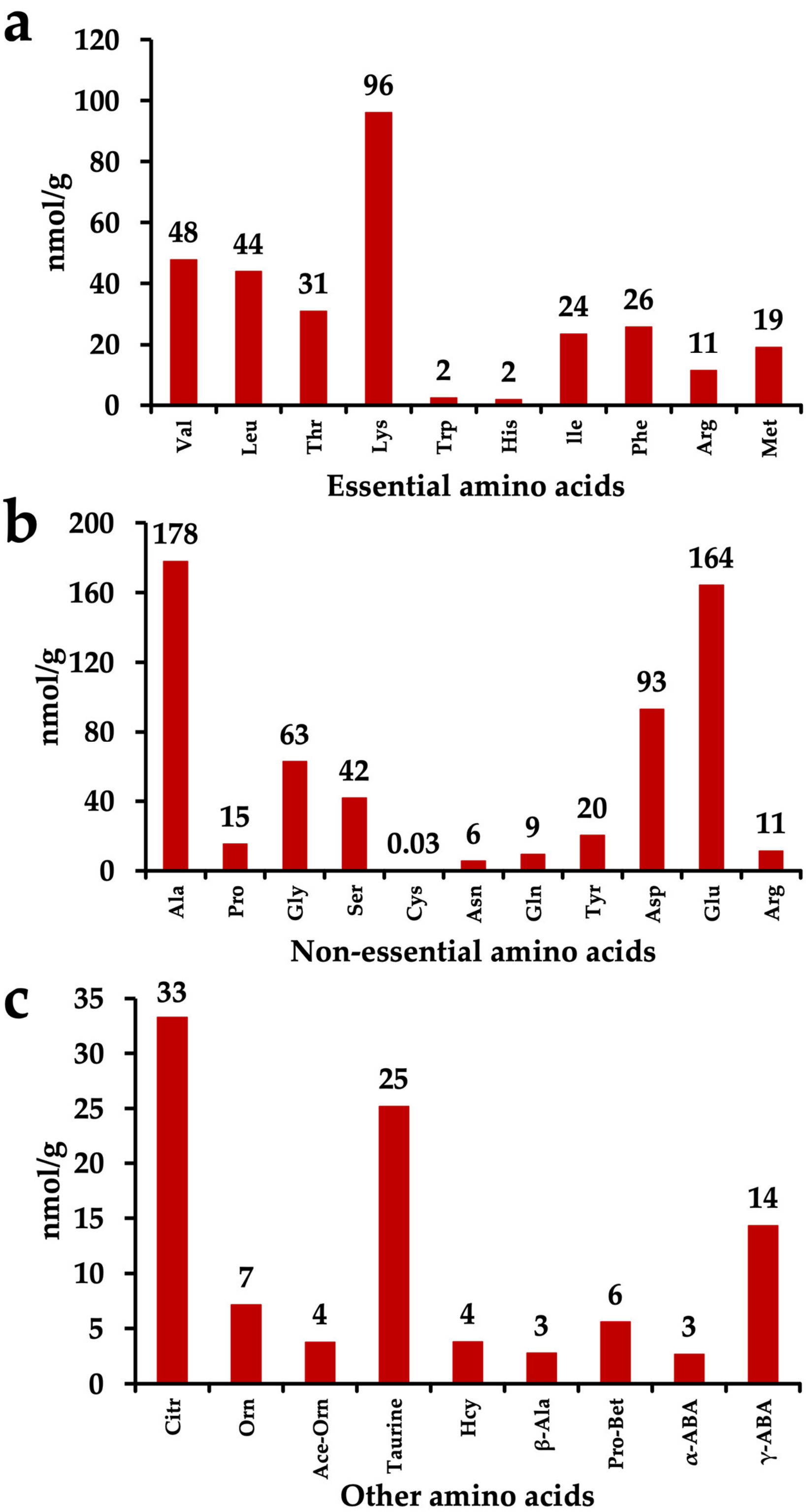

Supplementary Figure S2. Concentrations of amino acids (nmol/g of fresh feces) in dairy cows across treatments. **a**) Essential amino acids, **b**) Non-essential amino acids and **c**) Other amino acids. Treatments were: 1- Heat stress; 2- Heat stress with higher Vitamin D_3 and Ca (1,820 IU/kg and 1.5 % Ca); 3- Thermoneutral, pair-feeding. Treatments were allocated within two plots differing in basal concentrations of Vitamin E and Se (High: 200 IU/kg Vit. E and 1.2 mg/kg Se; Low: 20 IU/kg Vit. E and 0.3 mg/kg Se). Fecal metabolite data were obtained at day 14 of treatment initiation, and analyzed using a LC-MS/MS custom assay.

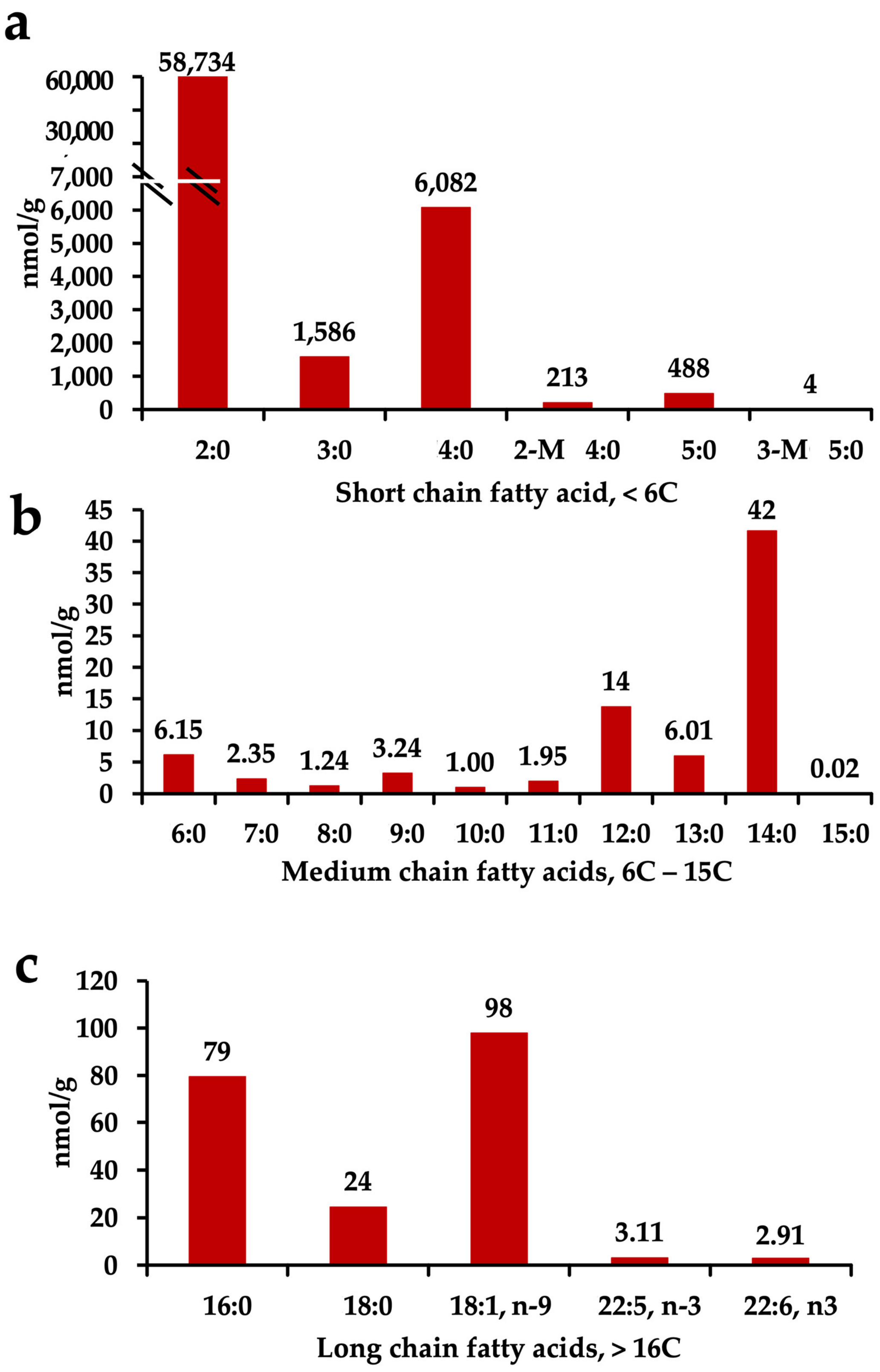

Supplementary Figure S3. Concentrations of fatty acids (nmol/g of fresh feces) in dairy cows across treatments. **a**) short-chain fatty acids (< 6C), **b**) Medium chain fatty acids (6C – 15C), **c**) Long chain fatty acids (> 16C). Treatments were: 1- Heat stress; 2- Heat stress with higher Vitamin D_3 and Ca (1,820 IU/kg and 1.5 % Ca); 3- Thermoneutral, pair-feeding. Treatments were allocated within two

plots differing in basal concentrations of Vitamin E and Se (High: 200 IU/kg Vit. E and 1.2 mg/kg Se; Low: 20 IU/kg Vit. E and 0.3 mg/kg Se). Fecal metabolite data were obtained at day 14 of treatment initiation, and analyzed using a LC-MS/MS custom assay.

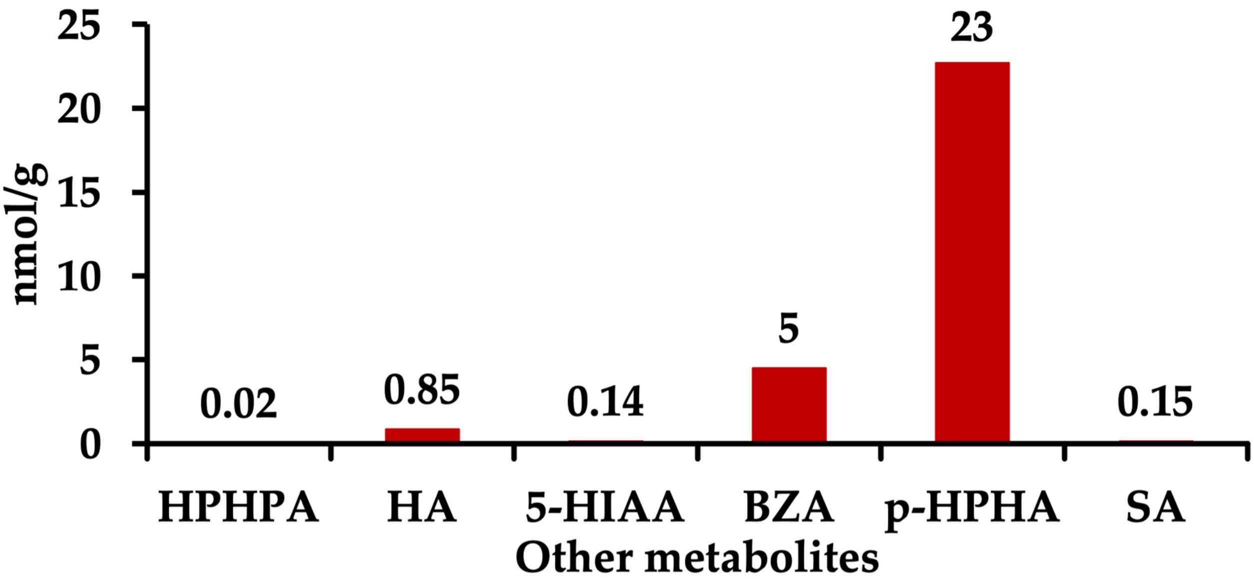

Supplementary Figure S4. Concentrations of various fecal metabolites (nmol/g of fresh feces) in dairy cows across treatments. Fecal metabolites concentration was obtained using a LC-MS/MS custom assay. 3-(3-hydroxyphenyl)-3-hydroxypropionic acid (HPHPA), hippuric acid (HA), 5-Hydroxyindoleacetic acid (5-HIAA), benzoic acid (BZA), p-Hydroxyindoleacetic acid (p-HPHA), and salilylic acid (SA). Treatments were: 1- Heat stress; 2- Heat stress with higher Vitamin D_3 and Ca (1,820 IU/kg and 1.5 % Ca); 3- Thermoneutral, pair-feeding. Treatments were allocated within two plots differing in basal concentrations of Vitamin E and Se (High: 200 IU/kg Vit. E and 1.2 mg/kg Se; Low: 20 IU/kg Vit. E and 0.3 mg/kg Se). Fecal metabolite data were obtained at day 14 of treatment initiation, and analyzed using a LC-MS/MS custom assay.

Supplementary Table S1. Metabolites concentration (nmol/g of fresh feces) in fecal samples of dairy cows using a LC-MS/MS custom assay. ND = not detected.

Metabolite	Mean	Std deviation	Minimum	Maximum
Vitamin B_1	15.4	6.09	0.45	28.4
Vitamin B_2	0.34	0.16	0.20	0.98
Vitamin B_3	0.03	0.00	0.03	0.03
Vitamin B_3-amide	0.05	0.00	0.05	0.05
Vitamin B_5	5.61	2.79	1.56	12.2
Vitamin B_6	0.060	0.00	0.06	0.06
Vitamin B_7	2.50	0.91	0.92	4.62
Vitamin B_9	0.036	0.02	0.01	0.10
Vitamin B_{12}	ND	ND	ND	ND
Vitamin C	0.009	0.00	0.01	0.01
Retinol	23.1	8.90	9.66	48.3
Vitamin D_2	ND	ND	ND	ND
25-HydroxyVitamin D_3	ND	ND	ND	ND
α - Tocopherol	20.3	14.3	3.93	60.6
$\beta + \gamma$ - Tocopherol	6.65	3.94	1.09	17.7
Δ - Tocopherol	0.59	0.77	0.01	3.03
Alanine	178	68.4	44.1	339
Arginine	11.4	8.06	2.36	46.5
Asparagine	5.571	1.42	2.69	8.79
Aspartate	93.1	51.6	6.39	235
Citrulline	33.3	14.18	11.25	64.5
Glutamate	164	89.1	29.1	504
Glutamine	9.35	5.11	3.18	28.8
Glycine	62.8	25.2	17.5	154
Histidine	1.89	2.68	0.07	10.9
Tyrosine	20.3	9.27	4.86	43.8
Valine	47.9	19.2	11.3	92.4
Isoleucine	23.5	10.8	4.95	47.4
Leucine	44.0	20.9	11.6	104
Lysine	96.1	39.2	23.8	179
Methionine	19.1	9.01	1.93	39.9
Ornithine	7.15	5.99	0.17	24.5
Phenylalanine	25.8	10.7	5.28	51.6
Proline	15.3	8.26	0.18	34.2
Serine	41.7	16.2	11.1	72.3
Threonine	30.9	12.7	8.52	56.1
Tryptophan	2.44	1.49	0.04	5.37
Methylhistidine	0.45	0.33	0.00	1.39

Choline	5.55	2.89	1.93	13.4
Sarcosine	ND	ND	ND	ND
Acetyl-Ornithine	3.80	3.12	0.39	12.4
Asymmetric dimethylarginine	ND	ND	ND	ND
Taurine	25.3	143	0.02	858
Trimethylamine N-oxide	ND	ND	ND	ND
Homocysteine	3.81	5.59	0.15	16.7
trans-OH-Proline	0.076	0.13	0.03	0.58
Alpha-aminoadipic acid	ND	ND	ND	ND
Carnosine	ND	ND	ND	ND
β - Alanine	2.80	3.66	0.42	19.0
Cystathionine	ND	ND	ND	ND
Proline-Betaine	5.60	1.75	2.36	8.97
Caffeine	ND	ND	ND	ND
α - Amino-N-butyric acid	2.69	2.83	0.02	11.0
γ - Aminobutyric acid	14.3	7.62	2.76	36.6
Anserine	ND	ND	ND	ND
Cotinine	ND	ND	ND	ND
Trigonelline	ND	ND	ND	ND
Acetic acid	58,733	14,244	36,600	87,600
Propionic acid	1,586	557	816	3,150
Butyric+Isobutyric acid	6,082	2,107	2,703	12,600
2-Methylbutyric acid	213	139	51.60	591
Valeric+Isovaleric acid	4,878	211	187	1,419
3-Methylvaleric acid	4.33	3.31	0.01	12.0
Caproic acid	6.15	3.44	0.01	14.8
2-Methylhexanoic acid	ND	ND	ND	ND
Heptanoic acid	2.35	0.86	0.62	3.78
Caprylic acid	1.24	0.45	0.28	2.08
Pelargonic acid	3.24	1.18	1.04	6.06
Capric acid	1.00	0.50	0.20	2.30
Undecylic acid	1.95	0.63	0.61	3.18
Lauric acid	13.8	5.37	3.15	23.4
Tridecylic acid	6.01	4.06	1.18	16.9
Myristic acid	41.6	24.5	13.35	120.9
Pentadecylic acid	ND	ND	ND	ND
Palmitic acid	79.4	56.0	0.02	196
Palmitoleic acid	ND	ND	ND	ND
Margaric acid	ND	ND	ND	ND
Heptadecenoic acid	ND	ND	ND	ND
Stearic acid	24.5	29.6	0.20	135
Oleic acid	97.9	50.3	33.6	260

Linoleic acid	ND	ND	ND	ND
Alpha-linolenic acid	ND	ND	ND	ND
Gamma-linolenic acid	ND	ND	ND	ND
cis-8, 11, 14-Eicosatrienoic acid	ND	ND	ND	ND
Eicosatetraenoic acid	ND	ND	ND	ND
Eicosapentaenoic acid	ND	ND	ND	ND
Docosapentaenoic acid	ND	ND	ND	ND
Docosahexaenoic acid	ND	ND	ND	ND
HPHPA[a]	0.015	0.06	0.01	0.37
Hippuric acid	0.847	0.44	0.03	2.04
5-Hydroxyindoleacetic acid	0.143	0.00	0.14	0.14
Benzoic acid	4.51	2.16	0.29	10.2
p-HPHA[b]	22.7	27.6	1.63	127
Salilylic acid	ND	ND	ND	ND

[a] 3-(3-hydroxyphenyl)-3-hydroxypropionic acid (HPHPA)

[b] p-Hydroxyindoleacetic acid (p-HPHA)
